# Comparative effect of polishing and coatings on the surface properties of 3D-printed denture-base resin and teeth: an in vitro study

**DOI:** 10.2340/biid.v13.46909

**Published:** 2026-09-30

**Authors:** Yousif A. Al‐Dulaijan, Alawiyyah Alsayed, Mashael Alhejji, Saja Alsuwailem, Jehad Almutiri, Soban Q. Khan, Abdulrahman A. Balhaddad, Shaimaa M. Fouda, Mohammed M. Gad

**Affiliations:** a Department of Substitutive Dental Sciences, College of Dentistry, Imam Abdulrahman Bin Faisal University, Dammam, Saudi Arabia; b College of Dentistry, Imam Abdulrahman Bin Faisal University, Dammam, Saudi Arabia; c Fellowship in Prosthodontics Program, College of Dentistry, Imam Abdulrahman Bin Faisal University, Dammam, Saudi Arabia; d Department of Dental Education, College of Dentistry, Imam Abdulrahman Bin Faisal University, Dammam, Saudi Arabia; e Department of Restorative Dental Sciences, College of Dentistry, Imam Abdulrahman Bin Faisal University, Dammam, Saudi Arabia

**Keywords:** Additive manufacturing, CAD-CAM, surface roughness

## Abstract

**Background:**

The surface properties of three-dimensional (3D)-printed resins remain an area requiring further improvement. Therefore, this study aimed to evaluate the effects of different polishing and coating protocols on the surface roughness and hardness of 3D-printed denture base resins (DBRs) and denture teeth.

**Methods:**

Two 3D printed resins (ASIGA and NextDent) for DBRs and denture teeth were used to fabricate disk-shaped (15 ×2 mm) specimens. Per resin, 96 specimens (48 for denture base and 48 for teeth) were divided into 4 groups (n=12) according to surface treatment: no polishing-coating (NP-C), conventional polishing (CP), Optiglaze (OG) coating, and PRIZMA seal (PS) coating. Surface roughness (Ra) and hardness were measured using a profilometer and Vickers hardness tester, respectively. The data was analyzed using one & two-way ANOVA, and post-hoc Tukey’s tests (α=0.05).

**Results:**

For both DBR materials, polished and coated groups showed a significant decrease (p<0.001) in Ra compared to the unpolished group (ASIGA: 1.6±0.36 µm; NextDent: 1.13±0.17 µm). Coated groups had lower roughness than the polished group (p<0.05). Similarly, for both printed tooth resins, the unpolished group recorded the highest Ra values and differed significantly from the polished and coated groups (p<0.001). CP showed significantly higher Ra than OG and PS (p<0.001), with no significant difference between the two coated groups. Regarding hardness, no significant differences were found among unpolished, polished, and coated DBR groups (p>0.05), except for the OG group, which showed significantly higher hardness than other groups (p<0.001). Between the two resins, NextDent showed significantly higher hardness than ASIGA only in the OG group (p=0.009). The same trend was observed for printed teeth, where OG coating yielded the highest hardness values.

**Conclusion:**

Surface roughness of 3D printed DBR and denture teeth decreased after polishing and coatings. Coating using OG resin improved the hardness of DBR and denture teeth.

## Introduction

The growing integration of computer-aided design (CAD) and computer-aided manufacturing (CAM) technologies in dentistry has driven the development of numerous technical and clinical protocols for the fabrication of various dental materials [1, 2]. Dental prostheses can be fabricated using CAD-CAM technology through two distinct approaches: subtractive manufacturing, in which a milling device shapes the prosthesis from polymethylmethacrylate (PMMA) blocks, or additive manufacturing, in which the prosthesis is built layer by layer through three-dimensional (3D) printing of acrylic resin [3, 4]. Additive manufacturing via 3D printing can produce complex geometries, fabricate multiple parts in a single run, and significantly reduce material waste [4].

Denture-base resin (DBR) and denture teeth are among the various materials used in 3D printing in dentistry and are among the most widely utilized [5]. The surface properties of both DBR and denture teeth play a major role in their clinical selection. Surface features such as hardness and roughness can influence strength, color alteration, water sorption, microbial colonization, and overall oral hygiene [6]. These surface properties can be affected by multiple factors, including the material’s chemical composition, layer orientation during printing, and surface polishing or coating applied after printing [7, 8]. Several polishing protocols have been reported in the literature for removable prostheses, such as mechanical, chemical, and surface-coating methods, which aim to improve the surface characteristics and esthetic appearance of DBR and denture teeth [9, 10].

Roughness is a critical physical property that can modulate the color stability and bacterial adhesion of dental materials. Materials with high roughness are subjected to color change and bacterial attachment, leading to esthetic and biological failure [11, 12]. Therefore, finishing and polishing the DBR and denture teeth are important to avoid such complications. The mechanical polishing process involves slowly removing rough layers by using materials with varying degrees of abrasiveness. To achieve smoothness and brightness in the finished surface, polishing tools such as pumice stones and pastes loaded with oxides are used, along with wheels, cones, rubbers, and silicone tips attached to electric motors [13, 14]. Another method for mechanical polishing is the use of chair-side polishing burs [1]. Chemical polishing can be achieved by immersion in a preheated jar at 75 ± 1°C filled with methyl methacrylate (MMA) monomer [15]. Compared with chemical polishing, mechanical polishing has been reported to be more efficient in reducing surface roughness, water sorption, and solubility in acrylic resin [9]. Another polishing method involves applying a surface-coating agent, such as a resin coating. This approach helps decrease surface porosity and irregularities, while also improving wear resistance and minimizing color changes [16].

Beyond surface roughness, 3D-printed resins also exhibit lower flexural strength and hardness compared to heat-cured resins [17]. Hardness describes a material’s capacity to withstand permanent surface deformation when subjected to an applied force, reflecting how well it resists indentation or surface damage under load [18, 19]. In acrylic resins, hardness indicates the polymer matrix’s susceptibility to degradation. As hardness decreases, the matrix deteriorates, increasing the risk of material fracture, microbial retention, and discoloration, affecting the longevity of the DBR [17]. In one investigation, an increase in DBR hardness was observed when a nano-filled glaze resin was applied [18].

As previously noted, the surface of 3D-printed DBRs and teeth must be optimally polished, as surface roughness is associated with increased staining, biofilm accumulation, patient discomfort, tissue inflammation, and secondary caries [19]. The surface properties of 3D-printed resins are directly influenced by the polishing technique employed [20]. It has been reported that surface roughness, contact angle, and color stability of 3D-printed resins designated for tooth fabrication are affected by different polishing protocols, and that combining protocols yields improved surface and color outcomes [20]. In contrast, studies investigating polishing protocols for DBRs have produced variable results, and no clear standardized protocol has yet been established [1].

To achieve an adequately polished surface, manufacturers of 3D-printed materials widely advocate surface glazing with various sealants or coating agents, in addition to sequential polishing steps [20, 21]. Since smooth and glossy surfaces are considered optimal for 3D-printed resins, various surface treatments have been proposed [1]. Among these, resin coating has been investigated in several studies [2, 18]. Beyond its effect on surface smoothness and gloss, coating has been shown to improve the mechanical behavior of 3D-printed resins by increasing hardness and wear resistance, thereby enhancing the longevity of denture base materials [18]. However, while coating can improve surface characteristics, polishing may yield superior outcomes, particularly with long-term clinical use [2]. Furthermore, it is well documented that glazing agents and surface sealants tend to degrade over time during clinical use [20, 21].

Given the established importance of polishing protocols for the clinical performance of both DBRs and denture teeth, further investigation is warranted to validate previously proposed protocols or identify optimal new ones [22]. This is further supported by the lack of a standardized polishing protocol for newly introduced 3D-printed resin materials used in removable prosthesis fabrication. Therefore, this study aimed to evaluate the most effective polishing and coating protocols for 3D-printed DBR and denture tooth resins. The null hypothesis states that polishing and coating protocols have no significant effect on the surface roughness or hardness of 3D-printed DBR or denture tooth resins.

## Materials and methods

G*Power software (version 3.1, Heinrich-Heine-Universität Düsseldorf, Düsseldorf, Germany) was used to calculate the sample size [23]. Based on a previous study [1], two-way Analysis of Variance (ANOVA) with two levels in the first factor and four levels in the second factor (numerator df = 3), a minimum sample size of nine specimens per group was required to detect a large effect size (*f* = 0.4) at a significance level of 0.05 and a statistical power of 0.8. However, the sample size was increased to 12 specimens per group to account for potential manufacturing defects or testing failures during the experimental procedures. 3D-printed DBR and 3D-printed tooth resin were tested from two manufacturers; the material details and printing parameters, along with the study flowchart, are summarized in Table 1 and Figure 1.

**Table 1:** List of materials, composition, and fabrication methods.

Material	Brand name/Shade	Composition	Fabrication method and machines
ASIGA	**Denture base:** Denture 3D+ NextDent B.V., Soesterberg, The Netherlands **Shade**: Pink	N/A	**Mixer:** LC-3D Mixer, NextDent, 3D Systems, Vertex Dental B.V., Soesterberg, The Netherlands. **Printer:** ASIGA MAX™. **Post-curing machine:** ASIGA Flash (ASIGA, Erfurt, Germany)
	**Teeth:** DentaTOOTH resin (Photopolymerized Methacrylate) used with an ASIGA MAX printer (ASIGA, Alexandria NSW 2015, Australia). **Shade**: A2	7,7,9(or 7,9,9)- trimethyl-4,13-dioxo- 3,14-dioxa-5,12- diazahexadecane-1,16- diyl bismethacrylate, tetrahydrofurfuryl methacrylate, diphenyl(2,4,6- trimethylbenzoyl) phosphine oxide	
NextDent	**Denture base:** DentaBASE ASIGA, Erfurt, Germany **Shade**: Light pink	Ethoxylated bisphenol A dimethacrylate, 7,7,9(or 7,9,9)-trimethyl-4,13-dioxo-3,14-dioxa-5,12- diazahexadecane-1,16-diyl bismethacrylate, 2-hydroxyethyl methacrylate, silicon dioxide, diphenyl(2,4,6-trimethylbenzoyl)phosphine oxide, and titanium oxide	**Mixer:** LC-3D Mixer, NextDent, 3D Systems, Vertex Dental B.V., Soesterberg, The Netherlands. **Printer:** NextDent 5100, 3D Systems, Vertex Dental B.V., Soesterberg, The Netherlands. **Post-curing machine:** LC-D Print Box (3D Systems, Vertex Dental B.V., Soesterberg, The Netherlands).
	**Teeth:** NextDent C&B MFH (Micro Filled Hybrid) resin (methacrylic oligomers, phosphine oxides) **Shade**: A1	7,7,9(or 7,9,9)-trimethyl-4,13-dioxo-3,14-dioxa-5,12-diazahexadecane-1,16-diyl bismethacrylate, 2-hydroxyethyl methacrylate, ethoxylated bisphenol A dimethacrylate, silicon dioxide, diphenyl(2,4,6-trimethylbenzoyl)phosphine oxide, and ethylene dimethacrylate	

**Figure 1:**
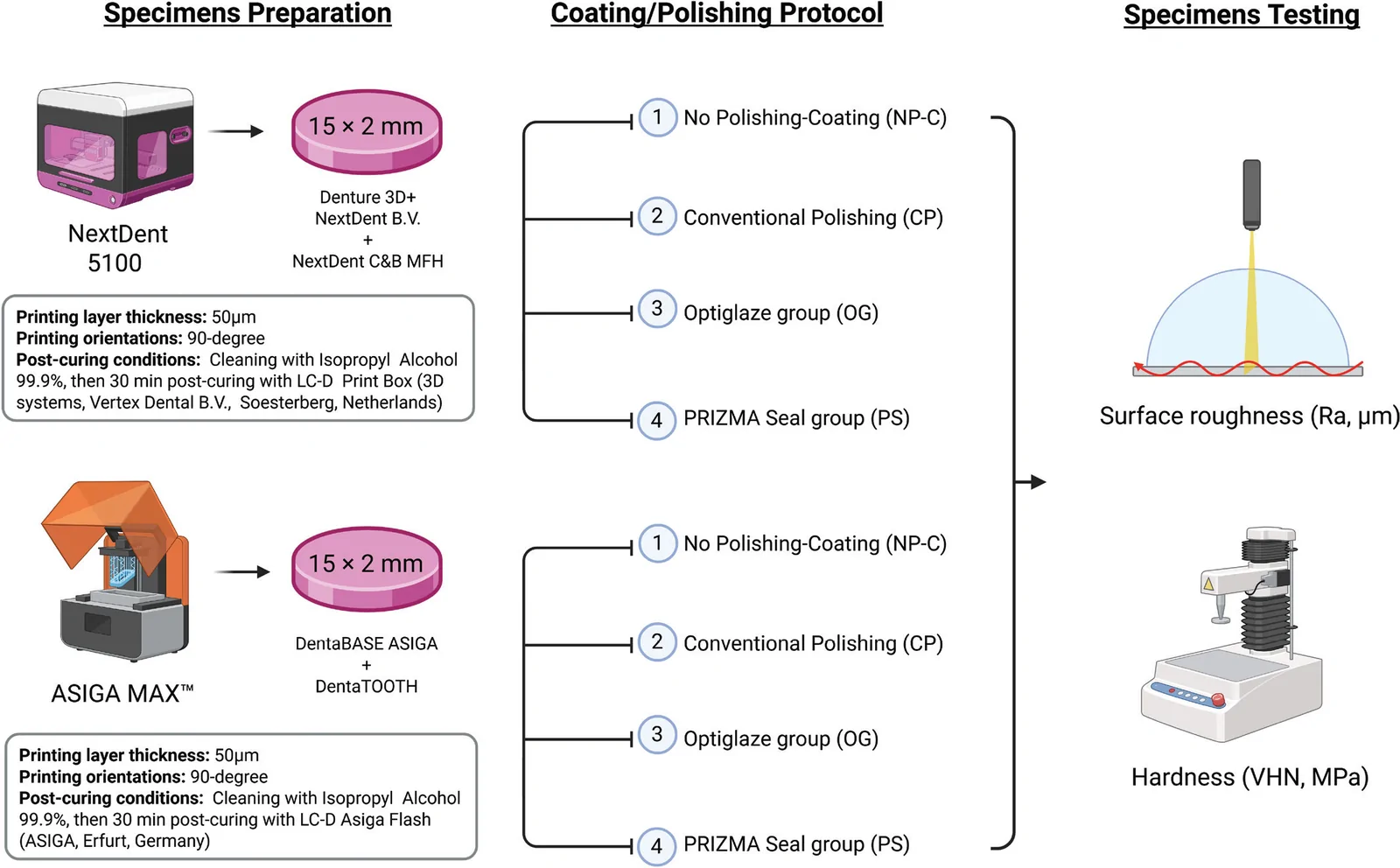
Schematic drawing of the study and list of printing parameters.

A disk-shaped specimen (15 × 2 mm) was designed using open-source CAD design (123D design, Autodesk, version 2.2.14, San Rafael, CA, USA), forming a Standard Tessellation Language (STL) file [24]. The STL file was imported to ASIGA MAX™ and NextDent 5100 printers. Specimen slicing, support position, and density were designed for each printer, with specimens printed at 0-degrees and a layer thickness of 50 µm. All printing processes were completed according to the manufacturer’s recommendation. After printing, the specimens were washed twice in an ultrasonic bath containing 99% isopropanol, with each cycle lasting 2 minutes [2]. For ASIGA, specimens were cured using ASIGA flash (ASIGA, Erfurt, Germany) and subjected to 4000 flashes (2000 flashes per specimen’s surface). For NextDent, specimens were placed into the LC-D Print Box (3D Systems, Vertex Dental B.V., Soesterberg, The Netherlands) and cured for 30 minutes at 60°C. After curing, the support structures were removed using a tungsten carbide bur mounted on a low‑speed rotary instrument.

The specimens were randomly distributed into four groups according to the polishing protocol. One group remained unpolished (NP-C), and the second group was polished by the conventional method (CP). The other two tested groups were coated with two resin-based coatings: Optiglaze (OG) and PRIZMA Seal (PS). A summary of the materials used and polishing protocols is presented in Table 2.

**Table 2:** List of surface treatments and different coating/polishing protocols.

Materials/code	Coating/Polishing protocols	Composition	Company
No Polishing-Coating (NP-C)	Finishing only; no additional polishing procedures were performed following support removal and water storage.		
Conventional Polishing (CP)	**First step:** polishing using pumice slurry on the brush attached to a grading motor machine (Red-Wing, Handler Mfg, Westfield, NJ, USA) at 3,000 rpm for 90 seconds.	Loose abrasives in liquid	Steribim Super, BEGO GmbH & Co KG; Bremen, Germany
	**Second step:** polishing with Universal Polishing Paste on lathe bristle brush at 3,000 rpm for 15 seconds.	Aluminum oxide (Al_2_o_3_) particles in paste	Ivoclar Vivadent AG; Schaan; Liechtenstein
Optiglaze group (OG)	**Application:** a single layer of OG clear was applied using a bristle brush. **Curing:** a UV chamber with a wavelength of 395–405 nm was used for 10 minutes.	Methyl methacrylate (MMA) (20-50%), diphenyl(2,4,6-trimethylbenzoyl)phosphine oxide as photoinitiator, nano-fillers, silicon dioxide, 5%−10% Titanium dioxide, 1%−5% and other additives	Optiglaze Clear, GC Corporation, Tokyo, Japan
PRIZMA Seal group (PS)	**Application:** a single layer of the resin was applied to the surface in one direction to avoid bubble formation. Then, it was left to dry for 15 seconds. **Curing:** a UV chamber with a wavelength of 395–405 nm was used for 10 minutes. **After curing:** it was left untouched for 5 minutes before removal.	Bisphenol A epoxylate dimethacrylate; 2,4,6-trimethyl-benzoyl-diphenylphosphineoxide; urethane dimethacrylate; tetrahydrofurfuryl methacrylate; amin	Makertech Labs, Sao Paulo, Brazil.

For surface roughness (Ra, µm) testing, a non-contact profilometer measured Ra for each specimen. After calibrating the device, three measurements were taken at the center of each specimen, spaced at least 0.5 mm apart. These measurements used a tracing length of 5.5 mm, a cut-off of 0.8 mm, and a stylus speed of 1 mm/s. The average Ra values were then calculated in micrometers [25]. For hardness testing, the hardness tester (FM-ARS 9000; Future-Tech Corp) was used to evaluate the Vickers Hardness (VHS) under a 25-g load for 10 seconds. For each specimen, three consecutive indentations spaced 500 μm apart were made and averaged to determine the Knoop VHN [25]. Surface roughness and hardness measurements were performed on the opposite surface from the one bearing the support structures, ensuring that removing the supports did not influence the evaluated surface properties.

Mean and standard deviation were computed as part of the descriptive analysis of the data. Normality of the data was tested using the Shapiro-Wilk test, and insignificant results from the test revealed that the data were normally distributed. Hence, parametric tests were used for inferential analysis of the data. The two-independent-samples *t*-test was used to study the difference between two averages in relation to the categorical variables with two categories. The one-way ANOVA test was used to compare more than two averages in relation to the categorical variables. The Tukey test was used for the pairwise comparison of means. A two-way ANOVA was used to examine the combined effects of material and coating on the tested properties. All *p*-values less than 0.05 were considered statistically significant.

## Results

Table 3 presents the mean values, standard deviations, and significance levels for surface roughness (Ra, µm) among the 3D‑printed denture base and tooth resin groups. For both DBRs, the conventional polishing and the two coated groups showed a significant decrease in Ra compared with the unpolished group (*p* < 0.001). The unpolished group exhibited the highest Ra values for ASIGA (1.60 ± 0.36 µm) and NextDent (1.13 ± 0.17 µm). Between the polished and coated groups, the coated groups demonstrated significantly lower Ra values than the polished group (*p* < 0.05), while no significant difference was found between the two coating protocols, OG and PS (*p* = 0.231). When comparing ASIGA and NextDent across all polishing/coating protocols, no significant differences were observed (*p* > 0.05). For the 3D‑printed tooth resins, the unpolished groups similarly showed significantly higher Ra values than the polished and coated groups (*p* < 0.001), with Ra values of 1.58 ± 0.30 µm (ASIGA) and 1.10 ± 0.20 µm (NextDent). Within the polished groups, CP exhibited significantly higher Ra values than the coated groups (OG and PS; *p* < 0.001), while OG and PS did not differ significantly. When comparing resin tooth materials according to polishing/coating protocol, significant differences between ASIGA and NextDent were observed in the NP‑C (*p* < 0.001) and CP (*p* = 0.039) groups, whereas no significant differences were found between the two resins in the coated groups OG (*p* = 0.739) and PS (*p* = 0.280).

**Table 3:** Two-way ANOVA for the combined effect of material and surface coatings for all tested properties for 3D-printed resins.

Properties	Resin	Source	Type III sum of squares	df	Mean square	*F*-value	*P*-value
**Roughness**	Denture base	Intercept	28.508	1	28.508	382.760	< 0.001*
		Resin	0.034	1	0.034	0.456	0.502
		Polishing/Coating	18.78	3	6.27	84.06	< 0.001*
		Resin * Polishing/Coating	20.243	7	2.892	38.828	< 0.001*
		Error	5.363	72	0.074		
		Total	54.114	80			
	Teeth	Intercept	22.746	1	22.746	565.033	< 0.001*
		Resin	0.122	1	0.122	3.03	0.086
		Polishing/Coating	17.45	3	5.81	144.5	< 0.001*
		Resin * Polishing/Coating	19.180	7	2.740	68.063	< 0.001*
		Error	2.898	72	0.040		
		Total	44.825	80			
**Hardness**	Denture base	Intercept	52814.601	1	52814.601	3406.112	< 0.001*
		Resin	68.06	1	68.06	4.39	0.043*
		Polishing/Coating	9164.59	3	3054.85	197.01	< 0.001*
		Resin * Polishing/Coating	9794.611	7	1399.230	90.239	< 0.001*
		Error	620.233	40	15.506		
		Total	63229.445	48			
	Teeth	Intercept	56246.736	1	56246.736	2654.554	< 0.001*
		Resin	9.75	1	9.75	0.46	0.501
		Polishing/Coating	7565.4	3	2521.8	119.01	< 0.001*
		Resin * Polishing/Coating	7609.424	7	1087.061	51.304	< 0.001*
		Error	847.551	40	21.189		
		Total	64703.711	48			

* *P* < 0.05 indicates statistical significance.

Table 4 summarizes the mean values, standard deviations, and significance levels for hardness (VHN) among the 3D‑printed denture base and tooth resin groups. For DBRs, no significant differences were detected between the unpolished, polished, and coated groups (*p* > 0.05), except for the OG group, which showed a significant increase in hardness compared with all other groups (*p* < 0.001). The highest hardness values were recorded for OG‑ASIGA (50.1 ± 4.0 VHN) and OG‑NextDent (64.0 ± 5.1 VHN). When comparing the two resins within each polishing/coating protocol, no significant differences were found except in the OG groups, where NextDent exhibited significantly higher hardness than ASIGA (*p* = 0.009). The hardness results for the 3D‑printed tooth resins followed the same trend as the denture base materials, with the OG coating producing the highest hardness values for ASIGA (57.7 ± 6.6 VHN) and NextDent (53.9 ± 7.1 VHN).

**Table 4:** Mean and SD of roughness (Ra, µm) of 3D-printed resins with different coating/polishing protocols.

Tested properties	Resins	Coating/Polishing protocols				*P*-value*
		NP-C	CP	OG	PS	
**Denture base**	ASIGA	1.6 ± 0.36^a^	0.54 ± 0.36^b^	0.167 ± 0.04^c^	0.15 ± 0.05^c^	< 0.001*
	NextDent	1.13 ± 0.17^a^	0.76 ± 0.53^b^	0.25 ± 0.15^c^	0.17 ± 0.07^c^	< 0.001*
	*P*-value **	0.22	0.218	0.218	0.912	
**Teeth**	ASIGA	1.58 ± 0.3^a^	0.31 ± 0.03^b^	0.18 ± 0.2^c^	0.22 ± 0.1^c^	< 0.001*
	NextDent	1.1 ± 0.2^a^	0.6 ± 0.4^b^	0.14 ± 0.03^c^	0.18 ± 0.1^c^	< 0.001*
	*P*-value **	< 0.001**	0.039**	0.739	0.280	

* *P* < 0.05 indicates statistical significance. *One-Way ANOVA results; different letters horizontally (per row) indicate significant differences between groups.

** *t*-test *P*-value results when comparing material per surface treatment; *P* < 0.05 is statistically significant.

Table 5 presents the interaction effects between resin type and polishing/coating protocol on surface roughness and hardness. The interaction between these two factors was statistically significant.

**Table 5:** Mean and SD of hardness (VHN, MPa) of 3D-printed resins with different coating/polishing protocols.

Tested properties	Resins	Coating/Polishing protocols				*P*-value
		NP-C	CP	OG	PS	
**Denture base**	ASIGA	25.8 ± 2.8^a^	27.2 ± 2.4^a^	50.1 ± 4.0^b^	24.8 ± 3.2^a^	0.003*
	NextDent	26.6 ± 2.5^a^	24.4 ± 2.6^a^	64.0 ± 5.1^b^	22.4 ± 2.4^a^	0.001*
	*P*-value**	0.699	0.093	0.009**	0.180	
**Teeth**	ASIGA	27.6 ± 2.6^a^	28.6 ± 3.7^a^	57.7 ± 6.6^b^	24.8 ± 1.8^a^	0.001*
	NextDent	27.3 ± 2.1^a^	29.3 ± 6.8^a^	53.9 ± 7.1^b^	24.6 ± 1.5^a^	0.001*
	*P*-value**	0.937	0.485	0.085	1.00	

* *P* < 0.05 indicates statistical significance. *One-Way ANOVA results; different letters horizontally (per row) indicate significant differences between groups.

** *t*-test *P-*value results when comparing material per surface treatment; *P* < 0.05 is statistically significant.

## Discussion

Although many studies have investigated the surface properties of 3D-printed DBR and denture teeth, no clear protocol or recommendations for polishing these surfaces have been reported. Therefore, this in vitro study was conducted to assess and compare the effect of polishing and coating protocols on the surface properties of 3D-printed DBR and denture tooth resins. The null hypothesis was partially rejected: all polishing and coating protocols improved the roughness of the investigated materials, but only one coating protocol using OP resin improved the materials’ hardness. Surface properties of removable dental prostheses play an important role in their esthetic success and patient satisfaction [26]. In clinical use, changes in surface properties affect color and surface stainability, as well as microbial adhesion and biofilm formation [27]. For optimal success, achieving a resin with a smooth surface and high hardness is important for increasing the clinical longevity of DBR and denture teeth [22]. Even with advanced technology, 3D-printed resins exhibit poorer surface integrity, which may limit their use compared with heat-polymerized and CAD-CAM-milled PMMA resins [28, 29]. Achieving a smooth prosthesis surface through polishing or glazing is essential to extend intraoral service life and minimize plaque accumulation [30]. Glazing involves the application of a light-polymerized clear resin coating that reduces surface porosity by penetrating the restoration surface to seal micropores and microdefects, thereby limiting microleakage [31, 32]. Prosthesis insertion often requires adjustments, such as occlusal and border adjustments, which necessitate guidelines for laboratory or chairside polishing [22].

In general, printed specimens showed higher roughness, attributed to the layer-by-layer printing process and the step-case effect of successive layers [33]. In addition, printing layer by layer may induce porosity, compromise surface integrity, and cause inadequate polymerization [34]. This finding is supported by a study, which shows that unpolished specimens have the highest Ra values. The Ra decreased with conventional polishing techniques, as polishing could mask the layering effect, resulting in a smooth surface. This finding is consistent with previous studies [1, 20], which reported that all polished specimens showed a significant decrease in Ra compared with their respective control groups. This confirms the importance of polishing and the need to optimize the polishing protocol for 3D-printed DBR and denture teeth. However, the recorded values with conventional polishing are still higher than the clinically recommended values of 0.2 µm. It has been reported that a roughness value greater than 0.2 µm is associated with an increased risk of bacterial colonization and plaque accumulation [35].

It is essential to note that in this study, when both coating agents were applied to DBRs, the roughness value was below the critical threshold of 0.2 µm. Similarly, in denture teeth, coating significantly reduced roughness compared to the conventional polishing technique, but only one coating technique was able to lower roughness by more than 0.2 µm. These findings highlight the importance of resin coating as a technique for improving the surface properties of DBRs and denture teeth. Coatings change the surface characteristics of resins, and this effect depends on the coat type, fluidity of the coat, and bonding to the resin surface [36]. These study findings agree with other investigations showing that the photopolymerized protective coating reduces the surface roughness of DBRs [37, 38]. Recent investigations found that polished 3D-printed denture bases, without any protective coating, exhibited higher surface roughness compared to milled, microwave-polymerized, and heat-polymerized denture base materials [22, 39]. This highlights the potential role of surface coating as an effective strategy to improve the smoothness of 3D-printed DBRs.

In this study, the performance of PS was investigated, as it has received limited attention in the literature. This study found that PS had the same effect as OG, reducing the roughness of the 3D-printed DBR. Both OG and PS reduced Ra to below or near the clinically acceptable threshold (0.2 µm), resulting in a smooth, glossy surface. This may be attributed to the coating layer, which flows over the resin surface, masking surface irregularities and producing a smooth and glossy appearance [29]. In addition, it was reported that the glaze can fill microstructures that resulted from finishing and polishing, such as micro-fissures and microdefects [16]. Another contributing factor may be the distribution and precipitation of nano-coat particles across the specimen surfaces, as these nanoparticles have the ability to fill surface pores and irregularities [29]. Similar findings to this study were observed in other investigations demonstrating that OG enhanced the roughness and hardness compared to conventional polishing [30, 40].

Considering the promising effect of glaze on the surface roughness compared to conventional polishing, the results should be interpreted with caution till further investigation proves these findings due to two main reasons. The first one is related to the aging of the glaze, which was not investigated in this study, while the second one is related to the thickness of the glaze over the specimen surface, which may affect the glaze quality [41]. The thickness of the coating layer is an important factor, as excessive thickness may compromise its retention. Therefore, coating thickness should be evaluated in future studies, either by incorporating coloring agents or by examining cross-sections using scanning electron microscopy (SEM). Based on the findings of the present study, it can be stated that glaze application to 3D-printed resin surfaces has a potential effect in reducing surface roughness.

Hardness is a surface property that reflects the material’s resistance to abrasion and scratching, thereby preserving the integrity of the material’s surface [42]. It was reported that polishing improves the hardness of the polished surface compared with the unpolished surface, and this improvement is due to the removal of printing-layer irregularities, which can improve polymer surface density and reduce porosity [42]. In the present study, conventional polishing did not significantly alter the hardness of 3D-printed DBRs or denture teeth. This may be attributed to the use of a conventional one-step polishing technique. Additionally, printing orientation may have played a role, as specimens were printed horizontally, exposing only the last printed layer as the specimen surface. This resulted in fewer surface irregularities compared to specimens printed at 45° and 90° building angles, where block-like protrusions evenly distributed across the surface were surrounded by depressions, leading to greater surface irregularities [43].

On the other hand, the OG coating improved the hardness of both resins, while the PS coating was comparable to the unpolished and polished groups. This improvement is mainly due to the presence of silicon and titanium dioxide in the resin formulation of the OG coating, which directly contributed to the significant improvements in hardness [22]. The results of this study are in agreement with previous studies [18, 44] showing that OG application increased the hardness of resins. Alouthah et al. investigated the effect of a photopolymerized nano-filled coating on DBRs and found that the application of a single layer of OG significantly increased the hardness of 3D-printed resin [18], consequently enhancing the longevity of denture base materials. Similarly, Choi et al. compared five different denture glazes and reported that the OG group exhibited significantly higher hardness values compared to all other groups [45]. Another study also found that the hardness of DBR was increased following the application of glaze resin [44]. This enhancement may be explained by the marked reduction in surface defects, particularly scratches, which are known to negatively impact surface hardness [6]. Supporting this, a previous study reported a significant increase in VHS values following glaze treatment of 3D-printed resins [46]. This outcome is consistent with the findings reported in the literature [18, 44, 47], and has been attributed to the presence of silicon dioxide (SiO_2_) nanoparticles incorporated within the glaze material, which reduce surface defects and consequently improve hardness [44].

Although surface coating is technically simpler and less time-consuming than mechanical polishing, it is worth noting that coatings have been shown to deteriorate over time, resulting in greater color changes compared to adequately polished surfaces [48, 49]. In the present study, coatings showed better performance in surface characteristics Ra and hardness with OG; however, the results of this study should be interpreted with caution due to the absence of other correlated factors and other stresses that could affect the results. The main factor is the absence of aging, such as thermal cycling and surface aging [41, 44, 50]. With Thermal aging, the coating acts as a barrier that decreases the amount of water sorption and improves the resin material itself by decreasing the plasticizing effect [41, 44, 51]. Nevertheless, their effect on coating may affect the bond of coating or may affect the properties of coating, such as shrinkage or crack-like formation [38]. Surface aging, such as the brushing effect, has a direct relation with the coating, affecting the surface integrity [18]. Therefore, further investigations testing coating aging are recommended to validate the selected protocols for 3D-printed resins designated for base and teeth fabrication.

Despite the interesting results obtained in this study, several limitations must be considered. Using only two materials, DBRs and teeth, does not represent all available 3D-printed resins in the market, and only using two glaze materials under controlled laboratory conditions is considered a limitation of the present study. In addition, the geometry of the specimens used in the study is not clinically relevant to DBR or denture teeth, as flat specimens were used for standardization. Moreover, the absence of in vitro aging (e.g. thermal aging and brushing) and the assessment of other parameters (e.g. color changes and water sorption) should be considered in future investigations. Finally, the absence of an oral environment, such as saliva, oral fluids, enzymes, and chewing stress, added limitations to the present study. Further research is needed to examine the influence of different glaze compositions and application methods on denture base and teeth resins under simulated oral conditions, including their relationship with discoloration and staining susceptibility. Such evidence would help optimize glazing techniques and improve the clinical performance and esthetics of denture prostheses.

## Conclusion

The conventional polishing protocol decreases the roughness of both DBRs and teeth but remains above clinically acceptable values. Regardless of the resin type, coating the 3D-printed denture base and the tooth resins reduced roughness to below the clinically acceptable value. Conventional polishing and glaze-resin coating, particularly OG coating, for 3D-printed DBR and teeth improved hardness. For both resins, OG resin coating could be recommended for DBRs and artificial teeth as a laboratory and chair-side polishing protocol. However, further investigations are required to demonstrate the durability of glaze application compared with the conventional polishing protocol.

## Data Availability

The data supporting this study’s findings are available from the corresponding author upon reasonable request.
